# Intraspecific mitochondrial gene variation can be as low as that of nuclear rRNA

**DOI:** 10.12688/f1000research.23635.1

**Published:** 2020-05-07

**Authors:** Tshifhiwa G. Matumba, Jody Oliver, Nigel P. Barker, Christopher D. McQuaid, Peter R. Teske

**Affiliations:** 1 Centre for Ecological Genomics and Wildlife Conservation, Department of Zoology, University of Johannesburg, Auckland Park, 2006, South Africa; 2 Department of Zoology and Entomology, Rhodes University, Grahamstown, 6140, South Africa; 3 Department of Plant and Soil Sciences, University of Pretoria, Hatfield, 0028, South Africa

**Keywords:** Purifying selection, diversifying selection, mismatch distribution, molecular dating, demographic history, population expansion

## Abstract

**Background:** Mitochondrial DNA (mtDNA) has long been used to date historical demographic events. The idea that it is useful for molecular dating rests on the premise that its evolution is neutral. Even though this idea has long been challenged, the evidence against clock-like evolution of mtDNA is often ignored. Here, we present a particularly clear and simple example to illustrate the implications of violations of the assumption of selective neutrality.

**Methods:** DNA sequences were generated for the mtDNA COI gene and the nuclear 28S rRNA of two closely related rocky shore snails, and species-level variation was compared. To our knowledge, this is the first study to use nuclear rRNA at this taxonomic level, presumably because this marker is assumed to evolve so slowly that it is only suitable for phylogenetics.

**Results:** Even though high inter-specific divergence reflected the faster evolutionary rate of COI, intraspecific genetic variation was similar for both markers. As a result, estimates of population expansion times based on mismatch distributions differed between the two markers by millions of years.

**Conclusions:** Assuming that 28S evolves effectively clock-like, these findings can be explained by variation-reducing purifying selection in mtDNA at the species level, and an elevated divergence rate caused by diversifying selection between the two species. Although these two selective forces together make mtDNA suitable as a marker for species identifications by means of DNA barcoding because they create a ‘barcoding gap’, estimates of demographic change based on this marker can be expected to be highly unreliable. Our study contributes to the growing evidence that the utility of mtDNA sequence data beyond DNA barcoding is limited.

## Introduction

Mitochondrial DNA (mtDNA) has long been a marker of choice for investigating concepts as diverse as estimating genetic diversity and effective population sizes, reconstructing species’ evolutionary histories, exploring spatial genetic subdivisions, and identifying cryptic species. All these methods assume that mtDNA variation conforms to the neutral model of molecular evolution ^1^, but violations of this premise have long been recognised ^2^. Over the past decades, much evidence has accumulated that mtDNA can be strongly affected by selective sweeps and background selection ^3– 6^. As a result, the usefulness of the marker in assessing genetic diversity ^7^ and exploring spatial genetic structure in continuously distributed populations ^8^ has been questioned, and corrections of the mitochondrial molecular clock that account for selection have been proposed ^9, 10^.

The implications of reduced genetic diversity at the species or population levels due to purifying selection has so far received little attention. When mutations in mitochondrial genes occur at fewer sites than expected under the neutral model ^11^, molecular dating of historical demographic events by means of evolutionary rate estimates that are typically based on inter-specific divergence ^12, 13^ will result in considerable underestimates. This is particularly likely because divergence between species can be strongly affected by diversifying selection that is driven by different environmental conditions ^14, 15^, resulting in a faster accumulation of mutations characterising each species than is expected under the neutral model.

Here, we explore this issue using mitochondrial and nuclear DNA sequence data from two common southern African snails of the genus *Afrolittorina*. The finding that data from two genetic markers whose mutation rates are assumed to differ by at least an order of magnitude have similar levels of intraspecific variation challenges the usefulness of mitochondrial DNA sequences for studying historical demographic changes.

## Methods

Specimens of the snails *Afrolittorina africana* and *A. knysnaensis* were collected at 34 sites throughout South Africa ( Table 1). DNA was extracted using the CTAB protocol ^16^, amplified with universal COI primers ^17^ and 28S primers LSU5 ^18^ and LSU1600 ^19^ following Williams *et al.* ^19^, and sequenced on an ABI PRISM 310 Genetic Analyzer (Applied Biosystems) using Big Dye Terminator v3.1 chemistry. Sequences were edited using MEGA7 ^20^, and 28S sequences were phased in PHASE v2.1.1 ^21^ using default settings. Genealogical relationships between COI haplotypes and 28S alleles were reconstructed using the median-joining algorithm ^22^ in popArt v1.7 ^23^. To explore the effect of using interspecific evolutionary rates to estimate species-level population size changes, we calculated population expansion time ^24^ using Arlequin v3.5 ^25^ using each marker’s slowest and fastest published rates for marine gastropods ( Table 2).

**Table 1.** Number of individuals of *Afrolittorina africana* and *A. knysnaensis* for which COI and 28S sequences were generated. 34 sites along the South African coastline were sampled, and these are arranged from west to east.

Site name	*A. africana*	*A. knysnaensis*
Port Nolloth	-	4
Groenriviersmond	-	5
Strandfonteinpunt	-	1
Lamberts Bay	-	5
Melkbosstrand	-	2
Paternoster	-	1
Yzerfontein	-	1
Rooiels	-	2
Cape Agulhas	-	2
Still Bay	1	2
Herolds Bay	-	2
The Wilderness	2	-
Sedgefield	1	1
Tsitsikamma	-	1
Jeffreys Bay	-	3
Cape Recife	-	8
Cannon Rocks	6	4
Bushmans River	3	4
Port Alfred	7	4
Fish River	6	-
Hamburg	2	-
Gqunube	4	2
Haga-Haga	8	10
Dwesa	6	-
Hluleka	2	6
Port St Johns	5	6
Port Edward	8	2
Ramsgate	4	4
Park Rynie	2	-
Mhlanga	4	-
Ballito	3	-
Sheffield	12	-
Zinkwazi	3	-
Mission Rocks	4	-

**Table 2.** Estimates of population expansions of the two species of *Afrolittorina* under the sudden expansion model. The moment estimator τ is equal to 2ut, where u equals 2 µk (μ is the mutation rate and k is the length of the sequence), and *t* is the time of expansion in million years (my).

Species	τ	Marker	μ (%.my ^-1^)	*t* (my)
*Afrolittorina knysnaensis*	2.00	COI	0.50 ^1^	0.40 (0.00 – 0.41)
			2.60 ^2^	0.07 (0.00 – 0.08)
	3.25	28S	0.01 ^1^	32.1 (18.5 – 61.3)
			0.05 ^2^	6.41 (3.69 – 12.3)
*Afrolittorina africana*	2.50	COI	0.50 ^1^	0.50 (0.30 – 0.79)
			2.60 ^2^	0.10 (0.06 – 0.15)
	2.75	28S	0.01 ^1^	27.1 (19.1 – 51.5)
			0.05 ^2^	5.42 (3.81 – 10.3)

^1^Malaquias & Reid 2009; ^2^Williams & Reid 2004. A generation time of one year was used.

## Results

**Figure 1.**
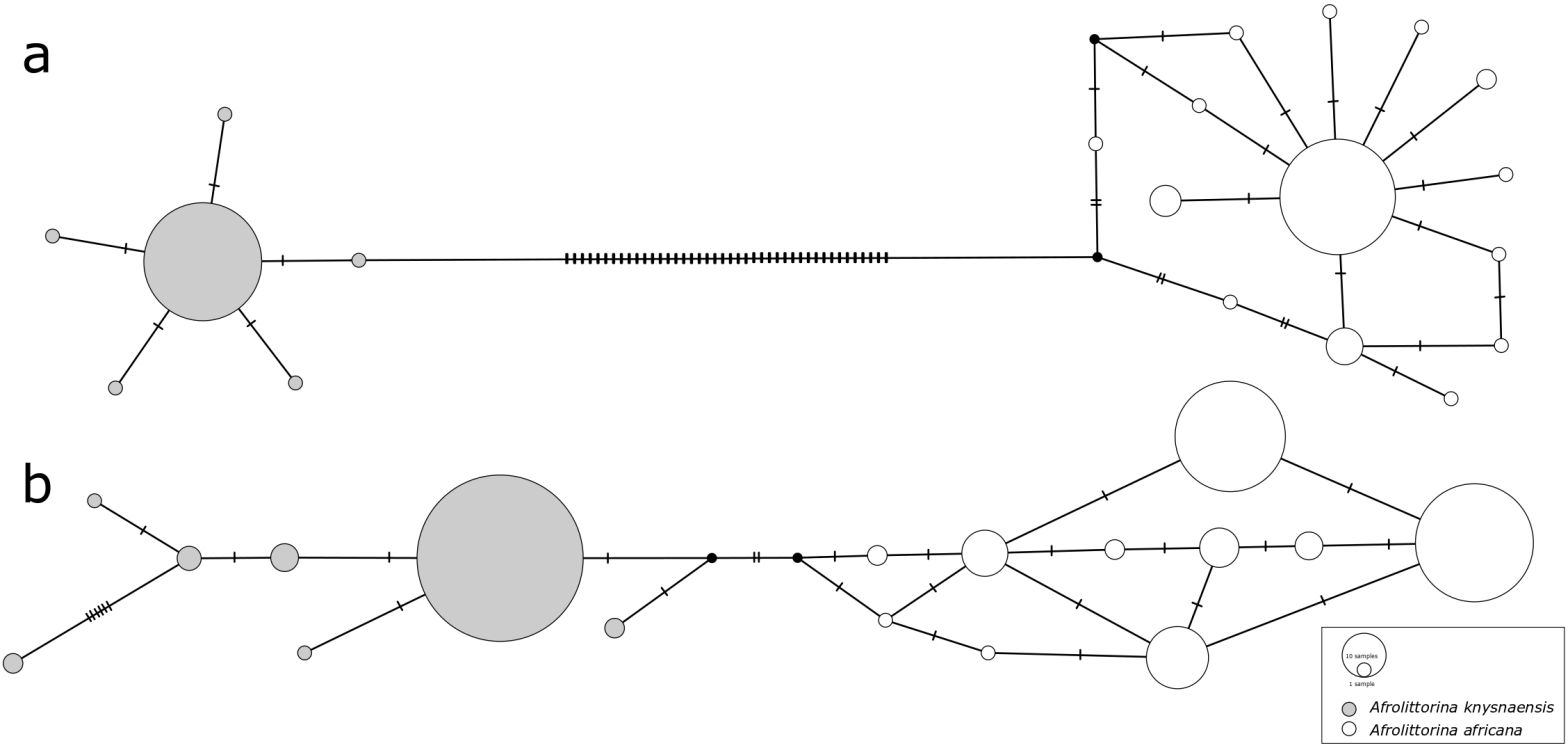
Median-joining haplotype networks constructed from **a**) COI sequences and **b**) 28S rRNA sequences of *Afrolittorina knysnaensis* (grey) and *A. africana* (white). Low intra-specific variation and high inter-specific variation of COI potentially illustrate purifying and diversifying selection, respectively. The size of circles is proportional to the frequency of each haplotype, cross-bars represent nucleotide differences, and black dots are missing haplotypes not found in the samples.

Species-specific genetic clusters reconstructed from COI sequences were highly distinct ( Figure 1a), with a minimum number of 44 nucleotide differences between the two species’ most closely related haplotypes. In contrast, differentiation between 28S sequences ( Figure 1b) was an order of magnitude smaller (4 differences).

In contrast to the high inter-specific differentiation between COI haplotypes, intra-specific genetic differentiation was comparatively low for this marker, and similar to that of 28S. In *A. knysnaensis*, six COI haplotypes and seven 28S haplotypes were found, while the maximum differentiation between the COI haplotypes was only two nucleotide differences, but 10 for 28S. The number of haplotypes was greater for *A. africana*, where 14 were found for COI and 10 for 28S. Maximum nucleotide differences for this species were seven in the COI network and five for 28S.

The practical implications of two markers with very different evolutionary rates based on inter-specific divergence having similar levels of intraspecific variation are illustrated in Table 2. Using published rates, estimates of population expansion times were more than an order of magnitude greater for the 28S data than for the COI data.

## Discussion

The usefulness of the mtDNA COI gene to uncover overlooked biodiversity is undisputed because of the marker’s tendency to have a well-defined barcoding gap, as was found here. The two study species’ COI sequences were much more strongly differentiated than their 28S sequences, potentially reflecting diversifying selection as a result of adaption to different thermal environments. In contrast, there was comparatively little genetic variation at the intraspecific level for either marker, which is likely due to the commonly reported strong purifying selection acting upon the COI gene ^6, 9^.

Many researchers explore their mtDNA sequence data for additional information, but the selective forces that together create the barcoding gap ^26^ make its utility for other applications questionable ^7, 8^. In the present study, we have highlighted a largely unexplored problem that likely arises from selection effects in mtDNA data: the fact that demographic events using gene regions under variation-reducing purifying selection are dated using molecular clock calibrations affected by variation-increasing diversifying selection. The finding that intraspecific mtDNA variation can be as low as that of nuclear rRNA cautions against the continued use of mtDNA for exploring demographic trends by means of mismatch distributions or Bayesian skyline plots ^27^, a practice that continues to dominate the recent literature ^28– 32^.

In our opinion, it is time to discontinue the use of fixed mtDNA rates based on divergence dating of closely related taxa, such as the closure of the Central American Seaway to date phylogenies of marine species ^12, 13^ or the 2% rule in birds ^33^. The very large datasets generated using next-generation sequencing have considerable potential to facilitate more accurate dating by identifying nuclear markers that conform to the assumptions of the molecular clock but curiously, fixed rates based on mtDNA data are still being used to calibrate such datasets when no suitable fossil calibration points exist ^34^. A possible solution may involve the identification of a suite of neutral markers that can be used to assess divergence between the species used in the original molecular dating studies, and 28S rRNA may be a suitable candidate.

## Data availability

DNA sequences generated in this study were submitted to GenBank (COI accession numbers: MT331645–MT331814; 28S rRNA accession numbers: MT329760–MT330099).
