# Supplementary material for: Intraspecific mitochondrial gene variation can be as low as that of nuclear rRNA
Source: F1000Res. 2020 Aug 28;9:339. Originally published 2020 May 7. [Version 2] doi: 10.12688/f1000research.23635.2 (PMC7475959; doi:10.12688/f1000research.23635.2)
Supplement: Peer review history

# Peer review: Reviewer response for version 2

**DOI:** 10.5256/f1000research.28975.r70500

**Published:** 2020-09-04

**Authors:** Urusa Thaenkham, Abigail Hui En Chan

**Affiliations:** 1 Department of Helminthology, Faculty of Tropical Medicine, Mahidol University, Bangkok, Thailand

We understand what the authors explained in the response to each comment. We know many limitations remarked in their responses. However, the readers who read this report in the future should realize that it was prepared and published under limitation conditions.

Is the work clearly and accurately presented and does it cite the current literature?

No

If applicable, is the statistical analysis and its interpretation appropriate?

Not applicable

Are all the source data underlying the results available to ensure full reproducibility?

Yes

Is the study design appropriate and is the work technically sound?

No

Are the conclusions drawn adequately supported by the results?

No

Are sufficient details of methods and analysis provided to allow replication by others?

No

Reviewer Expertise:

Molecular systematics.

We confirm that we have read this submission and believe that we have an appropriate level of expertise to confirm that it is of an acceptable scientific standard.

# Peer review: Reviewer response for version 1

**DOI:** 10.5256/f1000research.26080.r68386

**Published:** 2020-08-12

**Authors:** Urusa Thaenkham, Abigail Hui En Chan

**Affiliations:** 1 Department of Helminthology, Faculty of Tropical Medicine, Mahidol University, Bangkok, Thailand

This study aimed to explore the genetic variation of two common southern African snails of the genus Afrolittorina. The main finding of this study is that intraspecific variations of nuclear 28S rDNA sequences are higher than such value from the mitochondrial gene in the snail populations. However, many points need to concern before acceptance. I listed the major points for revising the manuscript.

1. Introduction part, the introduction part seems to focus on the selection process in the population to increase/decrease the genetic variation accumulation in the mt genome. The authors did not emphasize on the research questions; for example, Why did you want to study in two snails of the genus Afrolittorina? Why did you use the 28S rDNA as another genetic marker? What is the research question or hypothesis of this study? What was the reason behind deciding to study on *A. aficana* and *A. knysnaensis*? Finally, what is the main objective of this work? Whether you aimed to study the genetic differentiation between species of the snail based on different kinds of markers or study on population genetic, the content of this study could not support both objectives. For example, if the authors want to present on comparing the genetic variations that came out from the COI gene and 28S rDNA gene, the analysis used in this study was not suitable, the haplotype network analysis may not fit enough for the genetic variations you want to get. If the author wants to analyze the population genetically, the weak point is the numbers of the snails collected? And why the authors decided to collect from the various sites? What is the hypothesis behind your sampling?   
2. I don't understand why the authors have to analyze the molecular clock by comparing different originated markers like COI from one of the protein-coding genes in the mitochondrial genome and LSU (RNA-specifying gene) from the nuclear genome. Of course, the rate of evolution acting on these two genomes is different.
3. If the authors considered in Figure 1, you would realize that 28S rDNA is not good enough for species discrimination between *A. aficana* and *A. knysnaensis* comparing with the COI gene. Only 2 nucleotide differences between those two snail species, while the nucleotide variation in the intra-specific level of *A. knysnaensis* is higher than that.
4. The authors didn't discuss the evidence in snails with regards to variation, which is the point that showed in the result. 
5. There is no analysis to estimate either positive or negative selection, but the authors discussed it as the condition forced on the snail populations. It becomes over speculation. 
6. The title of this study should mention snail species because the genetic variation patterns are various depending on the groups of organisms. 

Is the work clearly and accurately presented and does it cite the current literature?

No

If applicable, is the statistical analysis and its interpretation appropriate?

Not applicable

Are all the source data underlying the results available to ensure full reproducibility?

Yes

Is the study design appropriate and is the work technically sound?

No

Are the conclusions drawn adequately supported by the results?

No

Are sufficient details of methods and analysis provided to allow replication by others?

No

Reviewer Expertise:

Molecular systematics.

We confirm that we have read this submission and believe that we have an appropriate level of expertise to confirm that it is of an acceptable scientific standard, however we have significant reservations, as outlined above.

# Response

**Published:** 2020-08-19

**Authors:** Peter Teske

**Reviewer Report: Abigail Hui En Chan and Urusa Thaenkham, Department of Helminthology, Faculty of Tropical Medicine, Mahidol University, Bangkok, Thailand**

APPROVED WITH RESERVATIONS

COMMENT: This study aimed to explore the genetic variation of two common southern African snails of the genus Afrolittorina. The main finding of this study is that intraspecific variations of nuclear 28S rDNA sequences are higher than such value from the mitochondrial gene in the snail populations.

RESPONSE: The statement that “intraspecific variations of nuclear 28S rDNA sequences are higher than such value from the mitochondrial gene” is not entirely correct, as in A. africana, COI has more haplotypes, whereas in A. knysnaensis, 28S has more haplotypes. We specifically state that “intra-specific genetic differentiation was comparatively low for this marker [i.e., COI], and similar to that of 28S.”

COMMENT: However, many points need to concern before acceptance. I listed the major points for revising the manuscript. Introduction part, the introduction part seems to focus on the selection process in the population to increase/decrease the genetic variation accumulation in the mt genome. The authors did not emphasize on the research questions; for example, Why did you want to study in two snails of the genus Afrolittorina? Why did you use the 28S rDNA as another genetic marker? What is the research question or hypothesis of this study? What was the reason behind deciding to study on A. aficana and A. knysnaensis? Finally, what is the main objective of this work?

RESPONSE: This article is a Brief Report, which has very strict word limits. For that reason, it is not possible to provide lengthy explanations for the issues raised. Briefly, the original purpose of the research was to determine whether each species is divided into regional populations (which was not found). The purpose was not to compare intraspecific variation between two markers with very different evolutionary rates, as that is an unexpected finding, and essentially a novel discovery that is reported in this paper. To answer the additional questions, we would like to refer the reviewers to the original PhD thesis on which this paper is based, which is now included among the references, and which is accessible via the following link: http://vital.seals.ac.za:8080/vital/access/manager/Repository/vital:5588?site_name=GlobalView

COMMENT: Whether you aimed to study the genetic differentiation between species of the snail based on different kinds of markers or study on population genetic, the content of this study could not support both objectives. For example, if the authors want to present on comparing the genetic variations that came out from the COI gene and 28S rDNA gene, the analysis used in this study was not suitable, the haplotype network analysis may not fit enough for the genetic variations you want to get. If the author wants to analyze the population genetically, the weak point is the numbers of the snails collected?

RESPONSE: The aim of the study was not to analyse each population genetically. We assume here that the reviewers equate ‘populations’ with sampling sites, please see the following paper for an explanation why this is problematic: Waples & Gaggiotti (2006) Molecular Ecology 15:1419-1439. As neither species shows regional sub-structure (see also the PhD thesis mentioned earlier) and each species thus comprises a single ‘population’, it is possible to pool data from all sites for intraspecific genetic analysis. The number of samples collected (93 and 82) is actually large compared to other papers of this nature. For example, in their highly cited paper on the barcoding gap (whose detection depends on both intra- and inter-specific variation), Paulay & Meyer (2005) used an average number of 7.7 samples per cowrie species.  

The fact that neither species of Afrolittorina is genetically subdivided throughout its range is clearly important, and was removed from an earlier version of this article so as not to exceed the word count for Brief Reports. We have decided to briefly mention it in the last paragraph of the Introduction: “Here, we explore this issue using mitochondrial and nuclear DNA sequence data from two common southern African snails of the genus Afrolittorina that show no spatial genetic structure throughout their ranges.” We now also cite the PhD thesis for anyone interested in the biology of these snails.

COMMENT: And why the authors decided to collect from the various sites? What is the hypothesis behind your sampling?

RESPONSE: The original purpose of collecting samples from multiple sites that span much of each species’ range was to determine whether or not these species are genetically subdivided into regional populations, which was not found. However, this is clearly not relevant to the present study, which focuses on intraspecific variation, not genetic structure. Obtaining large numbers of samples from a few sites would be problematic as one cannot rule out that additional, genetically distinct ‘populations’ exist that were not sampled. Please note that Table 1 is not particularly important to understand this paper, and for that reason should not be the focus of a lengthy discussion related to sampling. We originally had it in an Extended Data section, but the editor requested us to move it to the main text.

COMMENT: I don't understand why the authors have to analyze the molecular clock by comparing different originated markers like COI from one of the protein-coding genes in the mitochondrial genome and LSU (RNA-specifying gene) from the nuclear genome. Of course, the rate of evolution acting on these two genomes is different.

RESPONSE: The difference in evolutionary rates between these two markers is crucial to highlight the potential role of variation-reducing selection in COI. Without understanding this, it is not possible to understand the take-home message of this paper. We essentially point out that the known rate differences between these two markers manifest at the inter-specific level. The novel finding of this study is that intra-specific levels of variation are surprisingly similar. To clarify, the statement “Of course, the rate of evolution acting on these two genomes is different” is only true between species, not within species.

COMMENT: If the authors considered in Figure 1, you would realize that 28S rDNA is not good enough for species discrimination between A. aficana and A. knysnaensis comparing with the COI gene. Only 2 nucleotide differences between those two snail species, while the nucleotide variation in the intra-specific level of A. knysnaensis is higher than that.

RESPONSE: The purpose of this paper is not to suggest that 28S is a marker that is suitable for species discrimination, particularly when compared to COI. As we state in the first sentence of the Discussion, “The usefulness of the mtDNA COI gene to uncover overlooked biodiversity is undisputed because of the marker’s tendency to have a well-defined barcoding gap, as was also found here.” It is not clear what the reviewers mean by “not good enough”, the two genetic clusters are clearly distinct, although inter-specific divergence is clearly much lower. The likely reason for this is that 28S evolves in a more clock-like fashion while COI is strongly affected by selective forces, and this is another important point to understand, as a marker that does not evolve in a clock-like fashion should not be used for intraspecific molecular dating. Please see the second paragraph in the Discussion: “In the present study, we have highlighted a largely unexplored problem that likely arises from selection effects in mtDNA data: the fact that demographic events using gene regions under purifying selection are dated using molecular clock calibrations affected by variation-increasing inter-specific divergence.” Given the slow evolutionary rate of 28S, minimal differentiation between species is expected. What is unexpected are the similar levels of intraspecific variation (see title).

COMMENT: The authors didn't discuss the evidence in snails with regards to variation, which is the point that showed in the result.

RESPONSE: The results report the simplest and most intuitive means of describing instraspecific variation: the number of haplotypes, and the maximum number of mutations between them. Several sentences in the Discussion deal with this, e.g. first paragraph: “In contrast, there was comparatively little genetic variation at the intraspecific level for both markers, which is likely due to the commonly reported strong purifying selection acting upon the COI gene.”

COMMENT: There is no analysis to estimate either positive or negative selection, but the authors discussed it as the condition forced on the snail populations. It becomes over speculation.

RESPONSE: The paper originally included a test for selection, but as it was not informative and exceeded the word limit for Brief Reports, it was removed. We used a McDonald-Kreitman test (McDonald & Kreitman 1991) in MKT (Egea et al. 2008) to test for selection in COI. Out of 58 mutations for the whole dataset, only one was non-synonymous. Although the test had the highest possible proportion of adaptive substitutions (alpha) of 1.0, it was non-significant (P = 0.57), supposedly because of the lack of non-synonymous polymorphism.

Further, recent genomic evidence indicates that tests comparing synonymous and non-synonymous mutations are not actually conclusive about whether or not selection has taken place, because synonymous sites may be affected by strong selection even though no amino acid changes have taken place (Lawrie DS et al. (2013) Strong Purifying Selection at Synonymous Sites in *D. melanogaster*. PLoS Genet 9(5): e1003527). Again, a detailed discussion of these issues would far exceed the word limit, and would not add much to the study. The simple dataset used here is clearly not sufficiently informative to conclude that purifying selection is present, and for that reason we have changed the earlier title “Purifying selection can reduce intraspecific mitochondrial gene variation to that of nuclear rRNA” ( https://www.biorxiv.org/content/10.1101/2020.03.31.017764v1) to the present one, which merely focuses on intraspecific variation rather than selection.

We nonetheless do not think that mentioning selection is this context is over-speculative. There is strong evidence that a finding of barcoding gaps in hundreds of studies is the result of selective forces, and we cite several important papers in this context, including two at the end of the following sentence in the Discussion: “In contrast, there was comparatively little genetic variation at the intraspecific level for both markers, which is likely due to the commonly reported strong purifying selection acting upon the COI gene.” In terms of finding a COI barcoding gap, our study is merely an additional example, but the finding that intraspecific variation-reducing selection can be so significant that COI shows levels of variation similar to a marker that evolves much more slowly is novel.

COMMENT: The title of this study should mention snail species because the genetic variation patterns are various depending on the groups of organisms.

RESPONSE: We respectfully disagree – the practice of including taxonomic information in the title is standard procedure in taxonomy journals, but it is undesirable in a multidisciplinary journal such as F1000Research. In general, it is well known that shorter titles that report results are more likely to get accessed ( https://www.ncbi.nlm.nih.gov/pmc/articles/PMC3351256/). In the present case, the snail species, their geographic structure, their biology etc. are far less important than are the two genetic markers, and anyone who does not work on littorinid snails may consider the paper irrelevant to their own work if species names are included in the title. However, the finding of this study is likely applicable to a wide range of organisms given the mounting evidence for variation-reducing selection in mtDNA, and giving the impression that it is unique to two rocky shore snails would be an obstacle to further investigation into this issue. To provide an example, had Stoeckle & Thaler 2014 mentioned in the title of their article “DNA barcoding works in practice but not in (neutral) theory” that these findings are based entirely on birds, we would probably not have cited their paper, even though it is highly relevant. For that reason, we believe that it should be sufficient to mention “rocky shore snails” in the Abstract.

# Peer review: Reviewer response for version 1

**DOI:** 10.5256/f1000research.26080.r66088

**Published:** 2020-07-20

**Authors:** Andrew G. Briscoe

**Affiliations:** 1 Natural History Museum, London, UK

This is an interesting manuscript addressing the methodological issues with the continued trend of using mitochondrial DNA sequence data to infer genetic variation and population structure. The results are based on a very limited data set, however, they are only intended to further illustrate the issues highlighted by the authors **. **Apart from that, the study does not require much improvement. It is well written and structured and the results support the conclusions and therefore suggest indexing the article once the following issues have been considered and adequately resolved;

 The authors claim that this is the first study to use 28S ribosomal RNA at this taxonomic level. Do they mean in snails, as this gene has been used extensively for phylogenetics and some population level analysis? More info is needed.

 Could the authors include a reference when stating that there is an order of magnitude of difference between the mutation rates of the two markers used in the study?

 Reference 29 is cited as an example of the continued use of mtDNA for exploring demography, however, that study uses the mitochondrial control region rather than sequence data Protein Coding Genes (PCGs). Is there evidence that the mutation rates of this mitochondrial locus is under the same kind of diversifying selection as PCGs? If not, then the authors should consider changing or removing this reference.

 In the discussion the authors refer to the different thermal environments of the two snail species used in the study. It would be useful if the authors could elaborate on this as very little background about the species being studied is provided.

 The authors state that they are assuming 28S evolves in a clock-like manner. Can they provide a references/evidence for this as it is quite a bold statement, which has implications on some of the conclusions being drawn.

Is the work clearly and accurately presented and does it cite the current literature?

Yes

If applicable, is the statistical analysis and its interpretation appropriate?

Yes

Are all the source data underlying the results available to ensure full reproducibility?

Yes

Is the study design appropriate and is the work technically sound?

Partly

Are the conclusions drawn adequately supported by the results?

Partly

Are sufficient details of methods and analysis provided to allow replication by others?

Yes

Reviewer Expertise:

Molecular biology, mitogenomics, phylogenetics.

I confirm that I have read this submission and believe that I have an appropriate level of expertise to confirm that it is of an acceptable scientific standard.

# Response

**Published:** 2020-08-19

**Authors:** Peter Teske

This is an interesting manuscript addressing the methodological issues with the continued trend of using mitochondrial DNA sequence data to infer genetic variation and population structure. The results are based on a very limited data set, however, they are only intended to further illustrate the issues highlighted by the authors. Apart from that, the study does not require much improvement. It is well written and structured and the results support the conclusions and therefore suggest indexing the article once the following issues have been considered and adequately resolved;

COMMENT: The authors claim that this is the first study to use 28S ribosomal RNA at this taxonomic level. Do they mean in snails, as this gene has been used extensively for phylogenetics and some population level analysis? More info is needed.

RESPONSE: We have conducted an extensive literature review on this issue, and have not found any comparable example. Even though intraspecific variation in 18S or 28S rRNA has been reported in various invertebrate taxa, upon closer inspection it becomes evident that this variation is either located in the more rapidly evolving ITS regions rather than in the rRNA, or the species studied have geographically distinct populations that may constitute cryptic species. However, as we cannot rule out the possibility that comparable examples exist, we have changed the sentence to: “Nuclear rRNA is not usually used to study intraspecific variation in species that are not spatially structured, presumably because this marker is assumed to evolve so slowly that it is more suitable for phylogenetics.”

COMMENT: Could the authors include a reference when stating that there is an order of magnitude of difference between the mutation rates of the two markers used in the study?

 RESPONSE: The paper already includes several references that have estimated mutation rates specifically for marine snails. Please see Table 2, where the 28S rates range from 0.01-0.05 %/myr, whereas the COI rates range from 0.26-0.50 %/myr. We now cite both references in the last paragraph of the Introduction.

COMMENT: Reference 29 is cited as an example of the continued use of mtDNA for exploring demography, however, that study uses the mitochondrial control region rather than sequence data Protein Coding Genes (PCGs). Is there evidence that the mutation rates of this mitochondrial locus is under the same kind of diversifying selection as PCGs? If not, then the authors should consider changing or removing this reference.

RESPONSE: This is a valid point, and while a signature of diversifying selection in the control region is likely because this marker is linked to the PCGs by virtue of being part of the same genome, it is clearly not under selection itself. For that reason, we have decided to remove this reference.

COMMENT: In the discussion the authors refer to the different thermal environments of the two snail species used in the study. It would be useful if the authors could elaborate on this as very little background about the species being studied is provided.

 RESPONSE: To comply with word count requirements for Brief Reports, we have removed more detailed descriptions of the species’ distribution ranges, morphology and physiology. In response to similar queries from the other reviewers, we now cite the PhD thesis on which the present study is based. This thesis not only provides information on the temperature-defined marine biogeographical provinces in which these species occur, but it also shows evidence for adaptive differences assessed using physiological experiments.

COMMENT: The authors state that they are assuming 28S evolves in a clock-like manner. Can they provide a references/evidence for this as it is quite a bold statement, which has implications on some of the conclusions being drawn.

RESPONSE: We have found no specific information on this, as the issue of selection on the molecular clock has primarily received attention in the context of mtDNA. However, we believe that the considerable difference in terms of genetic differentiation between species justifies such as statement. There is now strong evidence that the barcoding gap in COI is a result of selective forces, and the comparatively low level of genetic divergence based on 28S implies that similar selective forces are not acting on this marker. We have, however, made the statement in the Abstract less bold and have changed it to: “Assuming that 28S evolution is more clock-like…”.
